# Spinal manipulative therapy, Graston technique® and placebo for non-specific thoracic spine pain: a randomised controlled trial

**DOI:** 10.1186/s12998-016-0096-9

**Published:** 2016-05-16

**Authors:** Amy L. Crothers, Simon D. French, Jeff J. Hebert, Bruce F. Walker

**Affiliations:** Private practice of chiropractic, Geraldton, WA 6530 Australia; School of Health Professions, Discipline of Chiropractic, Murdoch University, South Street, Murdoch, WA Australia; School of Psychology and Exercise Science, Murdoch University, South Street, Murdoch, WA Australia; School of Rehabilitation Therapy, Faculty of Health Sciences, Queen’s University, Ontario, Canada

**Keywords:** Chiropractic, Spinal manipulation, Graston Technique®, Back pain, Thoracic spine

## Abstract

**Background:**

Few controlled trials have assessed the efficacy of spinal manipulative therapy (SMT) for thoracic spine pain. No high quality trials have been performed to test the efficacy and effectiveness of Graston Technique® (GT), an instrument-assisted soft tissue therapy. The objective of this trial was to determine the efficacy of SMT and GT compared to sham therapy for the treatment of non-specific thoracic spine pain.

**Methods:**

People with non-specific thoracic pain were randomly allocated to one of three groups: SMT, GT, or a placebo (de-tuned ultrasound). Each participant received up to 10 supervised treatment sessions at Murdoch University chiropractic student clinic over a 4 week period. The participants and treatment providers were not blinded to the treatment allocation as it was clear which therapy they were receiving, however outcome assessors were blinded and we attempted to blind the participants allocated to the placebo group. Treatment outcomes were measured at baseline, 1 week, and at one, three, six and 12 months. Primary outcome measures included a modified Oswestry Disability Index, and the Visual Analogue Scale (VAS). Treatment effects were estimated with intention to treat analysis and linear mixed models.

**Results:**

One hundred and forty three participants were randomly allocated to the three groups (SMT = 36, GT = 63 and Placebo = 44). Baseline data for the three groups did not show any meaningful differences. Results of the intention to treat analyses revealed no time by group interactions, indicating no statistically significant between-group differences in pain or disability at 1 week, 1 month, 3 months, 6 months, or 12 months. There were significant main effects of time (*p* < 0.01) indicating improvements in pain and disability from baseline among all participants regardless of intervention. No significant adverse events were reported.

**Conclusion:**

This study indicates that there is no difference in outcome at any time point for pain or disability when comparing SMT, Graston Technique® or sham therapy for thoracic spine pain, however all groups improved with time. These results constitute the first from a fully powered randomised controlled trial comparing SMT, Graston technique® and a placebo.

**Trial Registration:**

This trial was registered with the Australia and New Zealand Clinical Trials Registry on the 7^th^ February, 2008. Trial number: ACTRN12608000070336

## Background

Thoracic spinal pain is common with most occupational groups having 1-year prevalence around 30 % [1]. Commonly used treatment options for non-specific thoracic spine pain include manual therapies such as massage, mobilisation, and spinal manipulative therapy (SMT) [2], however there are no high quality studies evaluating these modalities. To date there are only two published randomised controlled trials whose primary aim was to assess the effectiveness of SMT on thoracic spinal pain [3, 4]. In the first study by Schiller [3] the author conducted a small study of 30 patients with ‘mechanical thoracic spine pain’ where SMT was compared to a sham comprising non-functional ultrasound. While patients in the SMT group reported lower pain intensity and greater lateral flexion range of motion immediately following the 2 to 3 week treatment period, there were no differences after 1 month. Concurrently, there were no between-group differences in McGill Pain and Oswestry Disability scores at any point of the trial.

In the second study by Lethola et al. [4], thoracic spinal manipulation and needle acupuncture led to similar outcomes as placebo electrotherapy in reducing pain in female patients with recent-onset mechanical thoracic spinal pain. This three arm study randomised 114 females aged 20–60 with thoracic spine pain (≤3-month duration) between the third and eighth thoracic vertebrae to receive a high-velocity thrust spinal manipulation, needle acupuncture, or placebo electrotherapy with intermittent suction. All interventions were provided by the same physiotherapist 4 times per week for 3 weeks. The study results showed small differences in pain reduction favouring manipulation 1-week post-intervention. However, these differences were not clinically important.

A third study was identified in a systematic review [5] of non-invasive interventions for musculoskeletal chest wall pain and thoracic pain, in this study [6] Stochkendahl et al. used a secondary outcome measure of severity of thoracic pain associated with their primary interest of acute chest wall pain. They studied 115 patients aged 18–75 years presenting to an emergency cardiology department in Denmark. Fifty-nine patients were randomised to receive a multimodal program of care provided by a chiropractor (up to 10 visits/4 weeks) including manipulation to the cervical and/or thoracic spine, combined with any or all of the following: joint mobilization, soft tissue therapy, stretching, stabilizing or strengthening exercises, heat or cold, and advice. There were 56 in the control group who received a single 15 min session of education provided by a chiropractor, which included reassurance and advice promoting self-management and individualized instruction on posture and home exercises to increase spinal movement or muscle stretch. While this study’s primary outcome was acute chest pain, data on thoracic pain was obtained and there were no differences between the groups at 4, 12 and 52 weeks.

The results of the trials above are similar to those found in systematic reviews of manual therapy for neck pain [7] and low back pain [8, 9].

For the purposes of this study we chose to evaluate SMT, Graston technique® (GT) [10] and a placebo treatment. The reasons for these choices are that SMT is a commonly used treatment worldwide [11] and GT is a popular soft-tissue technique in the United States and becoming more popular in other developed countries [12, 13]. GT is an instrument-assisted soft-tissue therapy involving the use of hand-held stainless steel instruments. The promoters of the GT [10] claim that the instruments resonate in the clinician’s hands allowing the clinician to isolate soft-tissue “adhesions and restrictions”, and treat them precisely. While there are two preliminary studies that show a) an increased blood flow with the use of GT [12] and b) improvements in shoulder ranges of motion among baseball players with its application [13] we are not aware of any high level evidence to support claims or the effectiveness of GT for spinal pain.

Given the lack of scientific evidence for the use of this modality and its apparent popularity for spinal pain a high quality trial was deemed necessary to determine efficacy.

Accordingly, we conducted a study to determine the efficacy of SMT and GT compared to sham therapy for the treatment of non-specific thoracic pain.

## Methods

The full protocol of this study has been published in free full text format elsewhere [14]. In summary, the study was a three-arm randomised, placebo-controlled trial, conducted between March 2008 and July 2009, comparing two treatment modalities to a sham intervention for people with acute or sub-acute thoracic spine pain. The therapy arms consisted of SMT and GT and the sham was non-functional ultrasound. Ethics approval was granted by Murdoch University Human Research and Ethics Committee (2007/274).

### Study sample and participant enrolment

The study was conducted at the Murdoch University Chiropractic student clinic in Perth, Western Australia. Participants were recruited using advertisements posted around the Murdoch University Campus, on local community boards and in newspapers.

Potential participants were screened with a detailed history and physical examination by a research assistant who applied the inclusion and exclusion criteria. Eligible participants were invited to enter the trial and asked to read and sign a consent form.

### Inclusion criteria

**Fig. 1:**
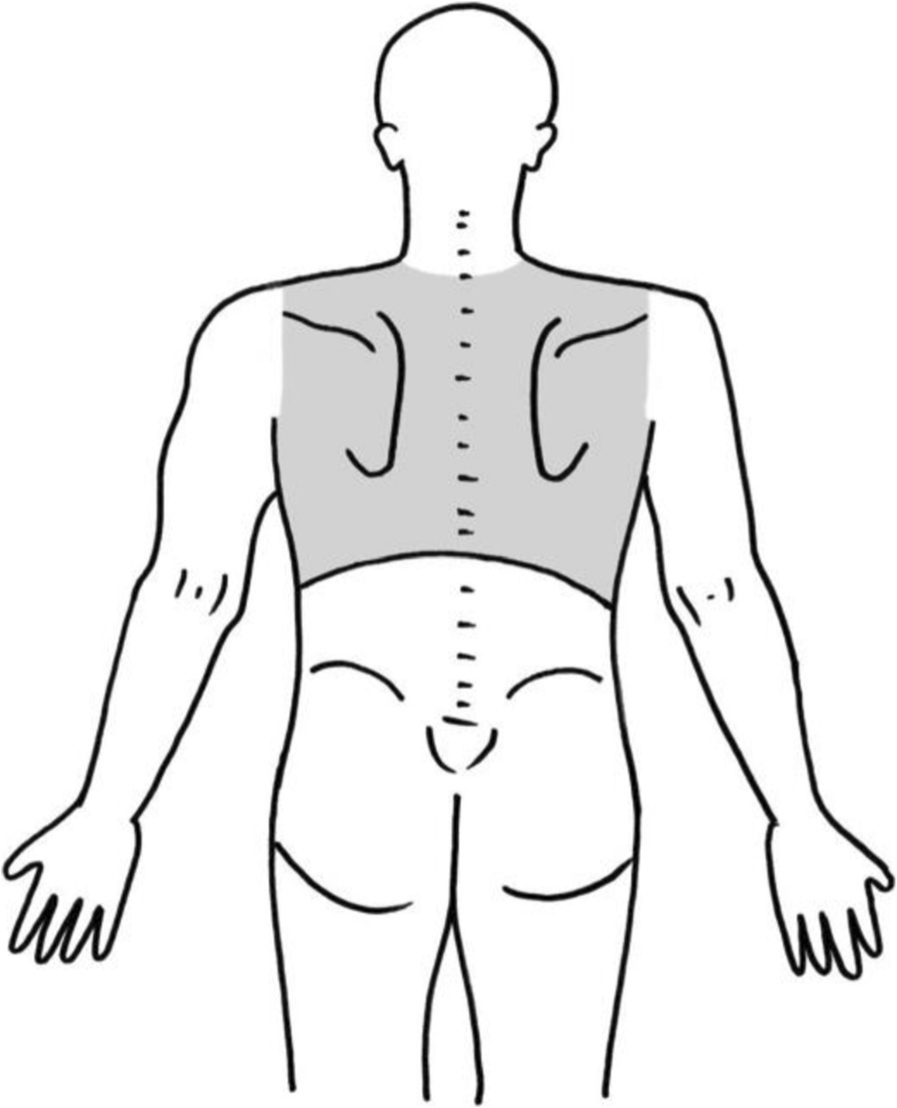
Shaded area defining the region of the thoracic spine where pain could be experienced for inclusion into the trial

People were included if they met the following criteria:

1. Age 18 years or older with non-specific thoracic spine pain of any duration, which was defined as pain in the region from T1 to T12 (Fig. 1) and complied with the descriptive classification by Triano et al. [15] (Table 1).

**Table 1:** Definition of non-specific thoracic spine pain [15]

- Midline back pain - for the purposes of this trial, the pain will be bound by the lateral margins of the thorax laterally and the trapezium superiorly	
- Non dermatomal referred pain difficult to localise	
- No signs of nerve root tension	
- No major neurological deficit	
- Pain with compression over the thoracic spine into spine extension	
- Reduced range of motion
2. A Visual Analogue Score (VAS) pain score of at least 2 out of 10 and an Oswestry Disability Index (ODI) score of greater than 15 % at baseline [16, 17].

### Exclusion criteria

People were excluded if they met any of the following criteria:

1. Had a contraindication to manual therapy including osteoporosis, thoracic fracture, spinal infection, neoplastic disorders, spondyloarthropathy, and clinical examination suggestive of frank disc herniation or generalised infection such as influenza.
2. Had a contraindication to Graston technique including neoplastic disorders, kidney infection, anticoagulant medication, rheumatoid arthritis, uncontrolled hypertension, thoracic fracture, osteomyelitis or generalised infection.
3. Had somatic conditions found on examination to refer pain to the thoracic spine from outside the defined area (including cervical zygapophyseal joints, muscles and discs).
4. Had an active history of visceral conditions referring pain to the thoracic spine including myocardial ischaemia, dissecting thoracic aortic aneurysm, peptic ulcer, acute cholecystitis, pancreatitis, renal colic, acute pyelonephritis
5. Had a current substance abuse problem.
6. Was not fluent and/or literate in the English language.
7. Was currently receiving care for thoracic pain from any other healthcare provider.
8. Could not commit to the full study protocol.
9. Was currently seeking compensation or had commenced litigation for thoracic spine pain.

### Treatment allocation

Randomisation occurred directly after baseline measures were taken and after the participant had been screened for inclusion. An online randomisation site, Research Randomiser [18], was used to generate treatment allocation. This online randomisation module is a web browser application that supports online randomisation of patients into healthcare trials. 150 random basis sequences without blocs were generated, and then placed in sequentially numbered, opaque, sealed envelopes and stored in a sealed box. As each participant entered the trial the next consecutive opaque, sealed envelope was given to the treating student and supervising clinician by the research assistant.

### Interventions and treatment

Participants were randomised to one of three treatment arms as follows:

1. Chiropractic group: a series of high velocity low amplitude chiropractic manual adjustments (SMT) to the thoracic spine were administered by a registered chiropractor or a final year chiropractic student under the direct supervision of a registered chiropractor. The thrust direction was at the discretion of the treating chiropractor or student.
2. Graston Technique group: Graston Technique was administered by final year chiropractic students who were certified in module one of the Graston Technique, under the direct supervision of a registered chiropractor who attended each consultation and in addition placed their hands on the anatomical regions involved;
3. Placebo group: participants received a session of de-tuned ultrasound administered by a final year chiropractic student, under the direct supervision of a registered chiropractor who attended each consultation and also placed their hands on the anatomical regions involved.

The reason for the random placement of hand near the painful area by the chiropractors for participants in groups 2 and 3 was designed to mitigate any lack of confidence in a student intern interaction and to provide a hands-on component to the therapy and make it more equivalent to the group 1 experience.

All treating students and chiropractors were briefed on the administration of the study and trained to show the same enthusiasm for all three treatment modalities. To minimise potential for attention effects, we standardised the delivery of therapy such that all sessions lasted 10–15 min, irrespective of treatment group. The administration of the various modalities and monitoring of progress is described in detail elsewhere [14] however, we aimed to administer 10 treatments of each of the “therapies” over 3–4 weeks to each participant.

### Change in inclusion criteria from the protocol

Initially, eligible participants were people with non-specific thoracic pain of less than 3 months duration. However, recruitment was slow so this was amended to also include participants with any duration of pain.

### Treatment allocation

Randomisation occurred directly after baseline measures were undertaken and the person had been screened for enrolment. A random number generator [18], was used to generate the treatment allocation sequence. Participant assignments were kept secure in sealed, opaque envelopes.

### Outcome measures and baseline data

Treatment outcomes comprised self-reported pain intensity with a 100 mm Visual Analogue Scale (VAS) and pain-related disability measured with a version of the Oswestry Disability Index (ODI) modified for patients with thoracic spine pain. These instruments are discussed in detail in the study protocol [14]. Outcomes were administered at baseline, 1 week after treatment commenced, upon completion of the 4-week intervention period and at three, six and 12 months.

A variation to the published protocol occurred with the decision to not obtain some baseline measures of race/ethnicity, education, household income, marital status, current employment status and general health status using the Short-Form Health Survey (SF-36). This was partly due to anticipated participant fatigue and that these extra baseline questions would likely put them off participating in the trial.

### Adverse effects

Participants were provided with a list of potential adverse effects in an information letter prior to giving to consent. Participants recorded information about adverse events in a log book.

### Blinding

The two outcome measures were self-administered instruments. Participants were given blank questionnaires in a package by a research assistant following their first treatment. Participants were instructed to complete the instruments at each assessment time point. After completion of the forms the participant posted them back to the Murdoch University Chiropractic Clinic. Research assistants remained blind to the outcome data for the entire study period. The participants and treatment providers were not blinded to the treatment allocation as it was clear that the groups were receiving different treatments. Participants in the placebo group were blinded to their placebo allocation until follow-up was complete at 12 months. Participants were surveyed for the adequacy of the placebo blinding at the end of the study.

### Sample size calculations

To calculate the sample size, we used the means of 23.9, 18.9 and 13.9 and assumed a standard deviation of 12.1 of the ODI derived in a study by Hoiriis et al. [19] in a similar chiropractic teaching setting. The clinical effect size used for the ODI was 10 % [17], alpha was set at .05. Recruiting 30 participants per group was calculated to provide 80 % power to identify between-group differences this large or larger. Sample size calculations using the VAS from the previous study resulted in a smaller n value for each group, therefore the ODI was used. We used 30 as a minimum requirement for each group.

### Data analysis

A researcher blinded to group allocation analysed the data. Statistical analyses were performed using IBM SPSS Statistics v21. The statistical analysis varied from the published protocol in the following way. Treatment effects were estimated using separate, random-intercept linear mixed models for each outcome variable. Time (1 week, 4 weeks, 3 months, 6 months, and 12 months) and treatment group (placebo, MT, Graston) were modelled as fixed effects. The hypothesis of interest was the time by group interaction which we further examined with pairwise comparisons of the estimated marginal means. We included the baseline outcome score as a covariate in each model. Consistent with the intention to treat principle, the linear mixed models estimated values for missing data based on available scores; therefore all participants randomised to a treatment group were included in the analyses of clinical outcomes. Alpha level was 0.05 for all analyses.

## Results

**Fig. 2:**
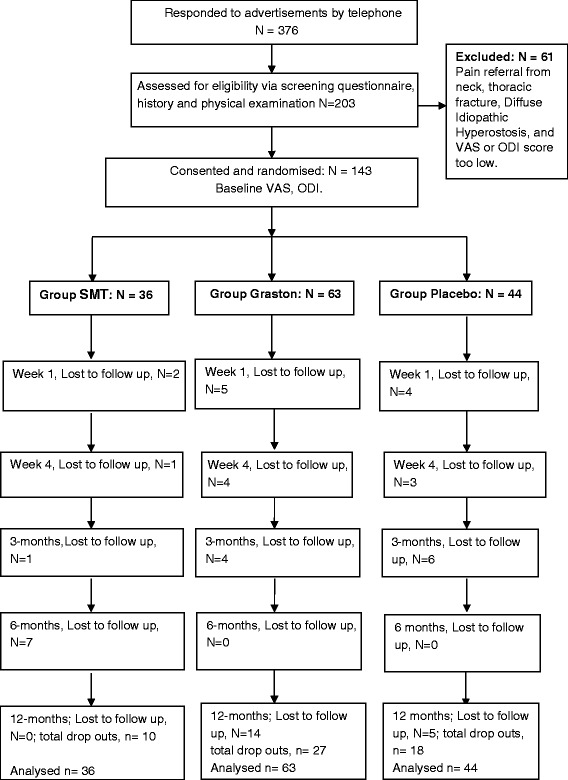
Flow chart of participants

The participant flow through the trial is presented in Fig. 2; 376 people responded to advertisements and after screening 143 patients were considered eligible for the study and were randomised to a study group. Demographic and clinical information at baseline is described in Table 2. There were no important differences in prognostic variables between the groups and it can be seen that our recruitment strategy resulted in an overwhelming number of participants with long standing pain.

**Table 2:** Baseline data for the entire sample and the three treatment groups

Variable	All (*N* = 143)	SMT (*N* = 36)	Graston (*N* = 63)	Placebo (*N* = 44)
Age (years)	45.8 (13.7)	44.4 (13.0)	44.8 (14.3)	48.5 (13.0)
Sex (% Male)	53.2	55.6	50.2	54.6
Pain (0-10 VAS)	5.6 (2.0)	5.5 (2.0)	5.7 (2.1)	5.5 (2.0)
Disability (0-100 ODI)	28.5 (10.4)	27.2 (10.2)	29.6 (11.1)	28.1 (9.9)
Pain duration (years)	9.2 (12.0)	9.0 (16.0)	8.2 (11.0)	10.9 (9.4)
Values are mean (standard deviation) unless otherwise indicated				

Of the total enrolled participants, there was a similar loss to follow up from each treatment group. Based upon results in Fig. 2, at 12 months the numbers lost to follow-up between groups compared to baseline, was SMT (28 %), Graston (45 %) and Placebo (41 %). Drop outs were unable to be contacted to determine the reason for loss to follow up.

Table 3 shows the pain (VAS) and disability (ODI) scores for the three treatment groups from baseline through all time points of follow up.

**Table 3:** Results of the intention-to-treat analysis comparing clinical outcomes between treatment groups

	Mean (SD) for each group			Adjusted mean difference between groups (95 % CI)		
	Graston	SMT	Placebo	Graston vs MT	Graston vs placebo	SMT vs placebo
Disability (0-100)*						
Baseline	29.6 (11.0)	27.2 (10.0)	28.1 (9.8)	-	-	-
1 week	22.6 (11.8)	22.3 (10.0)	23.7 (12.1)	-0.58 (-4.7,3.6)	-1.9 (-5.8,2.0)	-1.3 (-5.8,2.0)
1 month	18.1 (12.0)	19.8 (11.7)	21.5 (12.3)	-3.4 (-7.7, 0.9)	-4.5 (-8.6, -0.4)	-1.1 (-5.7, 3.6)
3 months	16.2 (13.1)	21.0 (14.3)	18.7 (15.0)	-4.6 (-9.5, 0.4)	-2.1 (-7.0, 2.8)	2.5 (-2.9, 7.9)
6 months	16.2 (13.1)	18.2 (14.2)	16.9 (14.1)	-1.9 (-6.9, 2.9)	-0.4 (-4.9, 4.2)	1.6 (-3.7, 6.9)
12 months	16.3 (13.5)	21.2 (16.0)	16.1 (16.3)	-4.8 (-10.5, 0.9)	-1.2 (-6.8, 4.4)	3.6 (-2.5, 9.7)
Pain intensity (0-10)^†^						
Baseline	5.7 (2.1)	5.5 (2.0)	5.5 (1.9)	-	-	-
1 week	4.7 (1.9)	5.1 (2.0)	4.7 (1.8)	-0.3 (-1.2, 0.5)	0.1 (-0.7, 0.8)	0.4 (-0.5, 1.2)
1 month	3.4 (1.9)	4.3 (2.0)	4.2 (2.4)	-1.0 (-1.9, -0.2)	-0.9 (-1.7, -0.1)	0.1 (-0.8, 1.0)
3 months	3.2 (2.6)	4.0 (2.2)	3.5 (2.3)	-0.8 (-1.8, 0.1)	-0.3 (-1.3, 0.6)	0.5 (-0.5, 1.5)
6 months	3.5 (2.5)	3.6 (2.2)	3.6 (2.5)	-0.4 (-1.4, 0.7)	-0.2 (-1.2, 0.8)	0.2 (-1.0, 1.3)
12 months	3.2 (2.3)	3.8 (2.4)	3.3 (2.5)	-0.8 (-1.9, 0.3)	-0.4 (-1.5, 0.7)	0.4 (-0.8, 1.6)

*Time by treatment group interaction *p* = 0.24

^†^Time by treatment group interaction *p* = 0.58

**Fig. 3:**
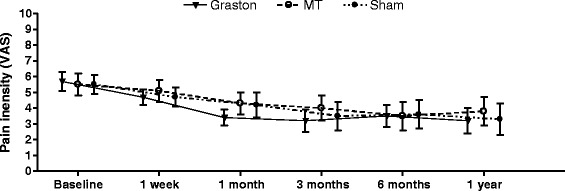
Mean group visual analogue scale pain scores over time with 95 % confidence intervals

**Fig. 4:**
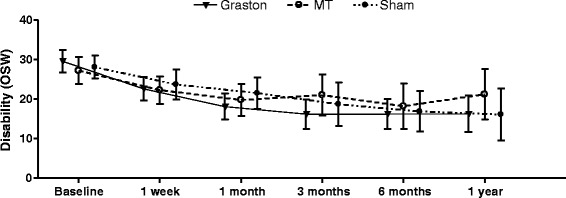
Mean group Oswestry disability scores over time with 95 % confidence intervals

Analysis demonstrates that there was no difference between the treatment groups for pain (Fig. 3) or disability (Fig. 4) at any time point.

Results of the intention to treat analyses revealed no time by group interactions, indicating no significant between-group differences in pain or disability at 1 week, 4 weeks, 3 months, 6 months, or 12 months (Table 3). There were significant main effects of time (*p* < 0.01) for both pain and disability indicating improvements in pain and disability from baseline among all participants (Table 3).

No significant adverse events were notified in this trial, however one participant reported increased pain after the placebo therapy. The participant dropped out of the study at this point and was lost to follow up.

### Blinding

Participants in the placebo group were asked at 12 months about the group to which they were allocated SMT, “other physical therapy” or just “other”. Only 8 participants responded to the question, 5 of whom thought they were in the SMT group and 3 in the “Other PT group”, none thought they were allocated to the “Other group” i.e. the placebo group.

## Discussion

Our study indicates that there is no difference in outcome at any time point for pain or disability when comparing SMT, Graston technique or placebo therapy for long standing thoracic spine pain, however all groups improved with time. These results constitute the first from an adequately powered randomised controlled trial comparing spinal manipulation, Graston technique and a placebo. It appears that our findings differ from the one other randomised trial [3] that reported SMT to be superior to sham for thoracic spine pain reduction, however that trial was inadequately powered and a Type II error was likely.

The results of our study are similar to those published for manipulation for low back pain [8] and for neck pain [7] where comparisons of manipulation to other modalities show only small treatment effects. The anatomy and biomechanics of the lumbar and cervical spines differ to that of the thoracic spine in that, among other things, the thoracic vertebrae are bound by ribs. However, our results suggest that these anatomical differences have not made any difference to clinical outcome after manual therapy is applied.

The strengths of this study lie in its randomised design and the inclusion of a sham or placebo arm for comparison of the active therapies involved. Another strength was the power of the study which was pre-determined and met by an initially adequate sample size.

Limitations to our study were the use of a modified Oswestry Disability Index (ODI) for the thoracic spine. We modified the original ODI [20] by replacing the words “low back pain” with “mid back pain” and doing this to the commonly used version where sexual difficulties had been removed. Otherwise the ODI was left intact. However it should be noted that the ODI was constructed for low back pain disability and as such may not be valid for mid back pain disability. Nevertheless there were no validated instruments to specifically measure thoracic spine pain related disability. Further validity studies should be undertaken to test this instrument for use in measuring thoracic spine disability. Even though dropout rates were similar between groups the lack of information regarding reasons for drop outs is also a limitation. This lack of information was caused by logistics and funding constraints that prevented us employing the human resources necessary to follow participants up. In addition, while we recorded the number of drop outs per group we did not record if a participant was pain free after less than 10 consultations and as such we are unable to report the average number of treatments per group or their range. Because of the size of the drop out rate treatment effect estimates beyond 3 months should be interpreted with caution.

Another possible limitation is the use of final year chiropractic interns to deliver the SMT and Graston therapy whose therapy outcomes may differ from more experienced clinicians. We did not record whether the intern or the practitioner delivered the treatment but is was under the supervision of the practitioner and had to be performed to their satisfaction. This was in accord with the published protocol. In addition, there is some evidence that therapist-related factors of increased experience and specialty certification status do not result in an improvement in patients’ disability associated with back pain [21]. All students had been certified in Stage I Graston therapy use by a certified Graston therapist. Regarding blinding of the placebo group we were unable to draw any conclusion on its success given the poor response to the question.

A final limitation occurred in the disproportionate numbers randomly allocated to the three groups, i.e. 36, 63 and 44. This imbalance resulted from the use of simple randomisation wherein each participant had an equal likelihood of being assigned to the groups. However, by chance an unequal number of individuals were assigned to each arm of the study and this may have adversely affected an optimum level of statistical power. Block randomization is a commonly used technique in clinical trial design to reduce bias and achieve balance in the allocation of participants to treatment arms, especially when the sample size is small [22]. In hindsight we should have used block randomisation as this would have resulted in equal group sizes.

The clinical implications and generalisability of this study are limited because while the results suggest that all of the methods tested provided benefit for chronic mid back pain this benefit included the placebo/sham arm. This apparent lack of effect may be due, at least in part, to the tendency to treat non-specific mid-back pain as a homogenous condition, rather than a heterogeneous collection of as yet undefined but differing conditions, some of which might respond and others that do not respond to a particular therapy. Research to identify diagnostic subsets within non-specific mid-back pain may be worthy and if successful, individual therapies such as manual therapy or Graston technique may be better directed.

## Conclusion

This study indicates that there is no difference in outcome at any time point for pain or disability when comparing spinal manipulative therapy, Graston Technique® or sham therapy for non-specific thoracic spine pain, however all groups improved with time. These results constitute the first from a fully powered randomised controlled trial comparing spinal manipulative therapy, Graston technique® and a placebo.

## References

[1] Briggs AM, Bragge P, Smith AJ, Govil D, Straker LM (2009). Prevalence and associated factors for thoracic spine pain in the adult working population: a literature review. J Occup Health.

[2] Hegmann K, Hegman KT (2011). Cervical and thoracic spine disorders. Occupational medicine practice guidelines Evaluation and management of common health problems and functional recovery in workers.

[3] Schiller L (2001). Effectiveness of spinal manipulative therapy in the treatment of mechanical thoracic spine pain: a pilot randomized clinical trial. J Manip Physiol Ther.

[4] Lehtola V, Korhonen I, Airaksinen O (2010). A randomised, placebo-controlled, clinical trial for the short-term effectiveness of manipulative therapy and acupuncture on pain caused by mechanical thoracic spine dysfunction. Int Musculoskelet Med.

[5] Southerst D, Marchand AA, Cote P, Shearer HM, Wong JJ, Varatharajan S, Randhawa K, Sutton D, Yu H, Gross DP (2015). The effectiveness of noninvasive interventions for musculoskeletal thoracic spine and chest wall pain: a systematic review by the Ontario Protocol for Traffic Injury Management (OPTIMa) collaboration. J Manip Physiol Ther.

[6] Stochkendahl MJ, Christensen HW, Vach W, Hoilund-Carlsen PF, Haghfelt T, Hartvigsen J (2012). A randomized clinical trial of chiropractic treatment and self-management in patients with acute musculoskeletal chest pain: 1-year follow-up. J Manip Physiol Ther.

[7] Gross A, Langevin P, Burnie SJ, Bedard-Brochu MS, Empey B, Dugas E, Faber-Dobrescu M, Andres C, Graham N, Goldsmith CH (2015). Manipulation and mobilisation for neck pain contrasted against an inactive control or another active treatment. Cochrane Database Syst Rev.

[8] Rubinstein SM, van Middelkoop M, Assendelft WJ, de Boer MR, van Tulder MW (2011). Spinal manipulative therapy for chronic low-back pain: an update of a Cochrane review. Spine.

[9] Walker BF, French SD, Grant W, Green S (2011). A cochrane review of combined chiropractic interventions for low-back pain. Spine.

[10] Graston Technique [http://www.grastontechnique.com/]. Accessed 18 Apr 2014.

[11] Hurwitz EL (2012). Epidemiology: spinal manipulation utilization. J Electromyogr Kinesiol.

[12] Portillo-Soto A, Eberman LE, Demchak TJ, Peebles C (2014). Comparison of blood flow changes with soft tissue mobilization and massage therapy. J Altern Complement Med.

[13] Laudner K, Compton BD, McLoda TA, Walters CM (2014). Acute effects of instrument assisted soft tissue mobilization for improving posterior shoulder range of motion in collegiate baseball players. Int J Sports Phys Ther.

[14] Crothers A, Walker B, French SD (2008). Spinal manipulative therapy versus Graston Technique in the treatment of non-specific thoracic spine pain: design of a randomised controlled trial. Chiropr Osteopat.

[15] Triano JJ, Hondras MA, McGregor M (1992). Differences in treatment history with manipulation for acute, subacute, chronic and recurrent spine pain. J Manip Physiol Ther.

[16] Kelly AM (2001). The minimum clinically significant difference in visual analogue scale pain score does not differ with severity of pain. Emerg Med J.

[17] Ostelo RW, de Vet HC (2005). Clinically important outcomes in low back pain. Best Pract Res Clin Rheumatol.

[18] Research Randomizer [http://www.randomizer.org/]. Accessed 18 Apr 2014.

[19] Hoiriis KT, Pfleger B, McDuffie FC, Cotsonis G, Elsangak O, Hinson R, Verzosa GT (2004). A randomized clinical trial comparing chiropractic adjustments to muscle relaxants for subacute low back pain. J Manip Physiol Ther.

[20] Fairbank JC, Couper J, Davies JB, O’Brien JP (1980). The Oswestry low back pain disability questionnaire. Physiotherapy.

[21] Whitman JM, Fritz JM, Childs JD (2004). The influence of experience and specialty certifications on clinical outcomes for patients with low back pain treated within a standardized physical therapy management program. J Orthop Sports Phys Ther.

[22] Efird J (2011). Blocked randomization with randomly selected block sizes. Int J Environ Res Public Health.

