# Erratum to: Spinal manipulative therapy, Graston technique® and placebo for non-specific thoracic spine pain: a randomised controlled trial

**DOI:** 10.1186/s12998-016-0111-1

**Published:** 2016-07-11

**Authors:** Amy L. Crothers, Simon D. French, Jeff J. Hebert, Bruce F. Walker

**Affiliations:** Private practice of chiropractic, Geraldton, WA 6530 Australia; School of Health Professions, Discipline of Chiropractic, Murdoch University, South Street, Murdoch, WA Australia; School of Psychology and Exercise Science, Murdoch University, South Street, Murdoch, WA Australia; School of Rehabilitation Therapy, Faculty of Health Sciences, Queen’s University, Ontario, Canada

## Erratum

The competing interest section of this article [1] is incorrect. The competing interests section should be as follows: BFW is Editor-in-Chief of Chiropractic & Manual Therapies, SDF is Deputy Editor-in-Chief, and JJH is an Associate Editor, however none of these authors had any involvement in the editorial process for this manuscript. The publisher apologises for any inconvenience caused.
